# Surgical Treatment of Maxillary Odontogenic Myxoma with Conservative Enucleation and Curettage: A Case Report

**DOI:** 10.3390/reports9030211

**Published:** 2026-07-03

**Authors:** Oscar Arturo Benítez-Cárdenas, Elhi Manuel Torres-Hernández, Miguel Angel Noyola-Frías, Ricardo Martínez-Rider, Marlen Vitales-Noyola

**Affiliations:** 1 Service of Oral and Maxillofacial Surgery, Regional High Specialty Hospital “Dr. Ignacio Morones Prieto”, San Luis Potosi 78290, Mexico; oscar.benitez@uaslp.mx (O.A.B.-C.); manuel.torres@uaslp.mx (E.M.T.-H.); manf001@uaslp.mx (M.A.N.-F.); 2 Department of Oral Surgery, Faculty of Dentistry, Autonomous University of San Luis Potosí, San Luis Potosi 78290, Mexico; rmrider@uaslp.mx

**Keywords:** odontogenic myxoma, conservative treatment, curettage, histopathology, recurrence

## Abstract

**Background and Clinical Significance**: Odontogenic myxoma (OM) is a rare benign neoplasm of the jawbones characterized by spindle-shaped cells embedded in a myxoid stroma. Despite its benign histological nature, it demonstrates locally aggressive behavior, significant invasiveness, and a high risk of recurrence. OM ranks as the third most common odontogenic tumor after odontoma and ameloblastoma. It affects both sexes and occurs more frequently in the mandible than in the maxilla, typically during the second to fourth decades of life. Macroscopically, OM is non-encapsulated, whitish-gray, and gelatinous. Radiographically, it usually presents as a radiolucent lesion with fine bony trabeculae, producing a characteristic “tennis racket” appearance. **Case Presentation**: We report a case of a 27-year-old male diagnosed with maxillary odontogenic myxoma measuring 2.3 × 1.7 cm. Clinical, radiographic, and histopathological findings were evaluated, and the lesion was treated conservatively by surgical enucleation and curettage. Results: The surgical procedure was completed without complications. Histopathological analysis confirmed the diagnosis of odontogenic myxoma. The patient showed satisfactory postoperative healing, and no evidence of recurrence was observed during a 10-month follow-up period. **Conclusions**: Although odontogenic myxoma is benign, its locally aggressive nature and recurrence potential require accurate diagnosis and appropriate management. Conservative treatment by enucleation and curettage may be effective for small, well-defined lesions, provided that careful long-term follow-up is maintained to monitor for recurrence.

## 1. Introduction and Clinical Significance

Lesions affecting the maxilla and mandible are frequently detected through imaging studies, particularly panoramic radiography, which remains a routine diagnostic tool in maxillofacial practice. Many jaw lesions are relatively rare and may remain clinically silent for prolonged periods, becoming symptomatic only after reaching considerable size or causing functional or aesthetic disturbances. Odontogenic cysts and tumors can occur in individuals of all ages, sexes, ethnicities, and socioeconomic backgrounds and are often discovered incidentally during routine radiographic examinations performed for unrelated reasons [1,2]. According to the 5th edition of the World Health Organization (WHO) Classification of Head and Neck Tumors (2022), odontogenic myxoma (OM) is defined as a benign neoplasm of odontogenic ectomesenchymal origin characterized histologically by spindle-shaped, stellate, or angular cells embedded within an abundant myxoid extracellular matrix rich in glycosaminoglycans [3]. Although benign, OM exhibits locally aggressive behavior, infiltrative growth, and a notable tendency for recurrence due to its lack of encapsulation and ability to permeate surrounding cancellous bone. Although the exact etiology of odontogenic myxoma (OM) remains controversial, it is widely accepted to arise from odontogenic ectomesenchyme, showing close structural similarities to the dental papilla, follicle, or periodontal ligament. Mechanistically, its development and locally aggressive behavior have been linked to the altered expression of extracellular matrix components, including glycosaminoglycans (mainly hyaluronic acid and chondroitin sulfate), and the up-regulation of matrix metalloproteinases (such as MMP-2 and MMP-9) which facilitate local bone resorption and tumor expansion. Additionally, recent molecular insights suggest that specific growth factors and anti-apoptotic signaling pathways may drive the proliferation of the characteristic spindle-shaped or stellate neoplastic cells within the prominent myxoid stroma [3].

Historically, the concept of myxoma dates back to 1863, when Rudolf Virchow introduced the term to describe tumors resembling the mucinous connective tissue of the umbilical cord [4]. The odontogenic variant was first described by Thoma and Goldman in 1947, establishing its distinct occurrence within the jaws [5]. Two clinical forms have since been recognized: the central (intraosseous) type, which is more common, and the peripheral (extraosseous) type involving soft tissues [3]. The pathogenesis of OM remains incompletely understood; however, it is widely believed to arise from odontogenic ectomesenchyme associated with tooth development, periodontal ligament mesenchyme, or dental papilla remnants. Alternative hypotheses suggest myxomatous degeneration of an odontogenic fibroma or persistence of embryonic mesenchymal tissue [6].

Clinically, OM typically presents as a slow-growing, painless swelling that can produce facial asymmetry, cortical bone expansion, tooth displacement, mobility, or malocclusion. Despite its indolent course, the lesion is capable of significant bone destruction and soft tissue infiltration. It predominantly occurs in tooth-bearing areas, with a higher frequency in the posterior mandible, although maxillary involvement is also reported. The tumor most commonly affects young adults, particularly during the second to fourth decades of life [7,8]. Radiographically, OM demonstrates variable features, ranging from unilocular to multilocular radiolucent lesions with well-defined or diffuse borders. Characteristic internal trabeculation patterns have been described as “honeycomb,” “soap bubble,” “tennis racket,” or occasionally “sunray,” reflecting residual bony septa within the lesion. The characteristic multilocular ‘honeycomb’ pattern is not pathognomonic and shares strong radiographical features with other osteolytic jaw lesions, requiring a strict differential diagnosis that includes multilocular ameloblastoma, odontogenic keratocyst (OKC), central giant cell granuloma (CGCG), and aneurysmal bone cysts [7]. Advanced imaging modalities such as computed tomography (CT) or cone-beam computed tomography (CBCT) may provide additional information regarding cortical perforation, extent of bone involvement, and proximity to adjacent anatomical structures.

The optimal management of odontogenic myxoma remains controversial. Treatment strategies depend on factors such as lesion size, anatomical location, biological behavior, patient age, and potential morbidity associated with surgery. Conservative approaches, including enucleation and curettage, are often preferred for small or well-circumscribed lesions, particularly in younger patients, to preserve function and minimize surgical morbidity [9]. More aggressive procedures, such as marginal or segmental resection with safety margins, may be indicated for large or recurrent tumors; however, evidence suggests that radical surgery does not necessarily eliminate the risk of recurrence [10]. Adjunctive treatments, such as the application of Carnoy’s solution without chloroform, have been proposed to chemically cauterize the bone cavity and eradicate residual tumor cells while avoiding carcinogenic effects associated with chloroform-containing formulations [11].

Given that OM frequently presents overlapping clinical and radiographic traits with other benign or malignant jaw pathologies, a preliminary incisional biopsy is an absolute requirement prior to definitive surgical planning. Undertaking a blind, single-step conservative enucleation accompanied by peripheral osteotomy without a prior histopathological diagnosis poses a significant clinical risk. An accurate preoperative histological identification ensures that the biological behavior of the lesion is fully understood, preventing undertreatment of aggressive tumors or unnecessary, mutilating overtreatment of non-invasive lesions, thereby optimizing the balance between complete therapeutic eradication and the preservation of crucial maxillofacial structures.

The present report describes the conservative surgical management of a maxillary odontogenic myxoma treated by enucleation and curettage, highlighting clinical, radiographic, and therapeutic considerations relevant to this uncommon but potentially aggressive odontogenic tumor.

## 2. Case Presentation

A 27-year-old male patient with a history of atopic dermatitis since birth, currently managed with topical emollients (Dexeryl^®^ Dermatological Expertise, Pierre Fabre Dermo-Cosmétique, Soual France), presented to our service. The patient reported a previous hospitalization in 2020 for severe COVID-19 associated with respiratory distress, requiring a five-day hospital stay. Additional clinical details are unavailable because the patient was treated at a different institution. However, according to national pandemic protocols at that time, hospitalized patients typically received high-dose corticosteroid therapy. During the clinical interview, the patient denied any other relevant medical or allergic history.

The current condition began approximately one year prior with progressive swelling in the right posterior maxilla, specifically in the vestibular gingival region. The patient reported previous root canal treatment of tooth #16, during which his private dentist suspected an infectious etiology and prescribed amoxicillin–clavulanic acid (875 mg/125 mg) for five days. Subsequently, an aspiration puncture was performed, but no purulent material was obtained. The patient was then referred to the Department of Oral and Maxillofacial Surgery at the Regional High Specialty Hospital “Dr. Ignacio Morones Prieto” in San Luis Potosí, Mexico.

Clinical evaluation revealed intermittent sharp pain localized to the right maxillary region. Intraoral examination showed no limitation in mouth opening. A well-defined erythematous swelling measuring approximately 1 × 2 cm was observed in the right maxilla. The lesion was tender on palpation, without local hyperthermia, with normal adjacent mucosa and no tooth mobility. Imaging studies, including cone-beam computed tomography (CBCT), demonstrated a well-defined hypodense lesion measuring 2.3 × 1.7 cm, extending from teeth #14 to #16 (Figure 1). An incisional biopsy was performed (Figure 2). Correlation of histopathological, radiographic, and clinical findings established the diagnosis of odontogenic myxoma (OM). Histopathological analysis now includes the presence of a loose myxoid stroma composed of spindle-shaped and stellate cells embedded in an abundant extracellular matrix, with delicate collagen fibers and absence of significant cellular atypia.

One week after diagnosis, the patient underwent surgical enucleation of the lesion under balanced general anesthesia using a conservative approach. The procedure was performed on an outpatient basis and lasted approximately two hours. Orotracheal intubation was achieved using a 6.5-mm endotracheal tube. After standard asepsis and antisepsis of the surgical field and placement of sterile drapes, local infiltration anesthesia with 2% lidocaine containing epinephrine was administered in the vestibular mucosa of the right maxilla. A linear incision was made using a #15 scalpel blade, followed by careful dissection to expose the lesion. Complete enucleation of the tumor was achieved, along with extraction of teeth #14, #15, and #16. Curettage of the surgical cavity was performed (Figure 3A), followed by irrigation to promote bleeding, suction, and primary closure using 4-0 Vicryl sutures (Figure 3B). No intraoperative complications occurred. A tissue specimen measuring 1.5 × 1.3 × 0.3 cm, reddish in color with soft myxoid areas, was submitted to the hospital pathology department. Histopathological examination confirmed the diagnosis of odontogenic myxoma.

Postoperatively, the patient was transferred to the recovery unit, where he remained conscious, hemodynamically stable, with vital signs within normal limits and adequate oxygen saturation on room air. He was discharged the same day with postoperative instructions and warning signs. Pharmacological management included clindamycin 300 mg every 6 h for seven days, oral paracetamol 1 g every 8 h for three days, ibuprofen 400 mg every 6 h for five days, and topical chlorhexidine 0.12% gel (Bexident^®^ ISDIN, Siegfied-Rhein, Barcelona, Spain) applied to the surgical site twice daily after tooth brushing for ten days.

At the one-week postoperative follow-up, the patient demonstrated satisfactory healing without complications. Six months after surgery, clinical examination and CBCT imaging revealed well-formed bone trabeculation and no evidence of recurrence (Figure 4). The patient remains under periodic surveillance. Ten months postoperatively (four months after the previous evaluation), repeat CBCT imaging showed a favorable outcome with no signs of tumor recurrence (Figure 5). The patient will continue follow-up visits every six months and subsequently on an annual basis.

In addition, the patient was recently recalled for a long-term follow-up at 24 months post-surgery. A control CBCT scan conducted at this follow-up milestone revealed complete, dense bone regeneration filling the previous maxillary defect, with well-defined trabecular reorganization and an intact cortical border. No clinical or radiographic signs of tumor recurrence were identified, demonstrating a stable long-term structural and functional outcome. Additionally, the chronological sequence of clinical presentation, diagnostic workup, surgical intervention, and long-term follow-up is summarized in Table 1.

## 3. Discussion

Odontogenic myxoma (OM) is a benign neoplasm of odontogenic ectomesenchymal origin characterized histologically by spindle-shaped, stellate, or angular cells embedded within an abundant myxoid extracellular matrix rich in glycosaminoglycans, as defined by the World Health Organization (WHO) Classification of Head and Neck Tumors [3]. Although histologically benign, OM exhibits locally aggressive behavior and infiltrative growth patterns, making clinical management challenging. Epidemiologically, OM is relatively uncommon among odontogenic tumors. In a classic study by Mosqueda-Taylor et al., which analyzed 349 odontogenic tumors, odontoma, ameloblastoma, odontogenic myxoma, adenomatoid odontogenic tumor, and calcifying odontogenic cyst were the most frequently observed lesions, with OM ranking among the most prevalent but still relatively rare entities [12].

Macroscopically, odontogenic myxomas typically present as non-encapsulated masses with a soft, gelatinous, whitish-gray appearance. The absence of a fibrous capsule allows the tumor to infiltrate surrounding cancellous bone in a permeative pattern, often extending beyond radiographically visible margins. This infiltrative growth pattern is considered a major factor contributing to local recurrence following conservative treatment [13,14].

The therapeutic management of OM remains controversial and ranges from conservative to radical surgical approaches. Conservative techniques include enucleation, curettage, peripheral osteotomy, and adjunctive chemical or cryosurgical therapies. Rocha et al. proposed an approach combining bone cavity curettage with cryotherapy using liquid nitrogen spray applied in three cycles of approximately one minute each, separated by thawing periods, to reduce residual tumor cells and recurrence risk [15]. In the present case, the lesion measured 2.3 × 1.7 cm and therefore met the criteria for conservative management suggested by Boffano et al., who recommend this approach for lesions smaller than 3 cm without evidence of aggressive behavior [16]. The lesion was relatively small and well localized, without evidence of extensive cortical destruction or involvement of adjacent anatomical structures, which supported a less aggressive surgical approach. Furthermore, conservative treatment was considered appropriate to minimize surgical morbidity and preserve surrounding tissues while maintaining favorable clinical outcomes for this patient. Favorable bone regeneration and absence of recurrence at 6- and 10-month follow-up evaluations support the effectiveness of this strategy in selected cases. In this specific patient, conservative enucleation and thorough curettage were preferred over radical resection primarily to preserve vital maxillo-facial structures, maintain the continuity of the maxillary arch, and minimize postoperative morbidity. Furthermore, adjunctive therapies, such as chemical cauterization with Carnoy’s solution or liquid nitrogen cryotherapy, were deferred due to the relatively small size (2.3 × 1.7 cm) and well-demarcated margins of the lesion, which allowed for an effective, complete mechanical debridement without risking chemical or thermal osteonecrosis of the adjacent thin maxillary cortical plates. This selective, less invasive approach balanced radical tumor removal with the preservation of optimal oral function and long-term quality of life.

More recently, Sato et al. emphasized that conservative treatment should include not only enucleation but also extensive curettage of surrounding apparently healthy bone, and in some cases peripheral osteotomy, to minimize residual tumor tissue while preserving adjacent vital structures and oral function [17]. Conversely, several authors advocate radical resection with safety margins of approximately 0.5–1.0 cm beyond the radiographic limits of the lesion for tumors demonstrating aggressive characteristics, such as cortical perforation, rapid growth, large size, or infiltration of surrounding soft tissues. Such approaches aim to reduce recurrence but are associated with increased morbidity and potential functional impairment [18,19].

Regarding the patient’s medical history, several comorbidities were present at the time of diagnosis, including atopic dermatitis, a recent COVID-19 infection managed with high-dose corticosteroids, and localized dental infections. While these conditions mandated a comprehensive systemic evaluation and careful surgical planning, there is currently no literature supporting a pathophysiological or causal link between these immunological, infectious, or pharmacological factors and the onset or progression of odontogenic myxoma. Therefore, they are considered concomitant findings that, although clinically significant for overall patient management, did not directly influence the primary diagnosis or biological behavior of the tumor.

Regarding the patient’s comprehensive long-term care, a definitive prosthetic rehabilitation plan for the secondary edentulous defect (teeth 14, 15 and 16) was thoroughly discussed with the patient. Given the optimal bone regeneration confirmed at the recent 24-month follow-up, a staged reconstruction involving secondary alveolar bone grafting and subsequent implant placement was highly recommended, and the patient was formally referred to an external oral rehabilitation specialist. However, since our hospital center does not provide advanced specialized prosthetic or restorative services, the definitive completion of this external treatment phase remains dependent on the patient’s compliance and financial feasibility, and cannot be strictly guaranteed or verified within our institutional protocol.

The clinical outcomes and limitations of conservative versus radical management in maxillary odontogenic myxoma have been documented in several recent case-citation reports. For instance, Sato et al. presented a long-term follow-up of an OM treated via conservative enucleation and curettage, reporting excellent bone regeneration and no recurrence over several years; however, they emphasized that the primary limitation of this approach is the absolute requirement for uncompromised, long-term radiographic monitoring, which can be difficult to guarantee if patient compliance fluctuates [17]. Similarly, Martins et al. reviewed a series of 13 cases undergoing conservative treatment, concluding that while enucleation preserves crucial structural architecture, its micro-infiltrative nature remains a constant risk factor for early recurrence, particularly in the maxilla where the thin, porous cortical plates facilitate silent tumor expansion into the maxillary sinus [18]. Furthermore, Li et al. highlighted that a major institutional limitation in retrospective series is the frequent loss of patient follow-up after the initial asymptomatic years, which underscores why conservative protocols must be carefully reserved for localized lesions under strict clinical surveillance [19]. These external findings align with our current case, demonstrating that while enucleation and thorough curettage minimize facial mutilation and optimize quality of life, they demand a rigorous long-term commitment to surveillance to counteract the inherent biological limitations of the technique [17,18,19].

Overall, the choice of treatment for odontogenic myxoma should be individualized, taking into account tumor size, location, biological behavior, patient age, and potential impact on function and aesthetics. Conservative enucleation combined with thorough curettage provides satisfactory outcomes for small, well-circumscribed lesions while preserving anatomical structures. Radical resection remains indicated for extensive, rapidly expanding, or recurrent tumors to achieve optimal local control [14,18]. Long-term follow-up is essential in all cases due to the tumor’s known recurrence potential.

## 4. Conclusions

Conservative surgical management with enucleation and thorough curettage represents an effective treatment option for small, well-circumscribed odontogenic myxomas, allowing complete lesion removal while preserving adjacent anatomical structures and oral function. Compared with radical resection, this approach is associated with lower morbidity, reduced functional impairment, and favorable postoperative bone regeneration, making it particularly suitable when the lesion shows no signs of aggressive behavior. Nevertheless, due to the tumor’s non-encapsulated and infiltrative nature, odontogenic myxoma carries a significant risk of recurrence. Therefore, prolonged clinical and radiographic follow-up is essential to detect potential relapse early and ensure optimal long-term outcomes.

## Figures and Tables

**Figure 1:**
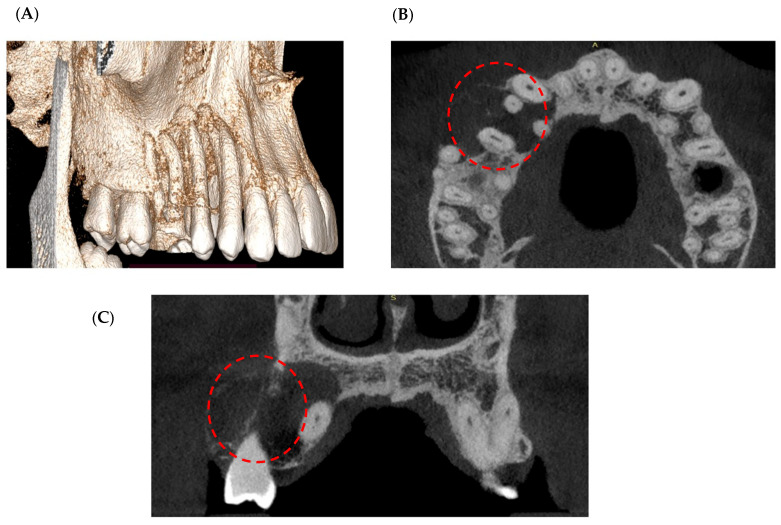
(**A**) Initial 3D reconstruction of CBCT showing the lesion area in the right maxilla. (**B**) Axial CBCT slice of the right maxilla revealing tumor lesion with cortical bone expansion. (**C**) Coronal CBCT slice. Red dashed lines indicate the specific areas of clinical interest and lesion margins.

**Figure 2:**
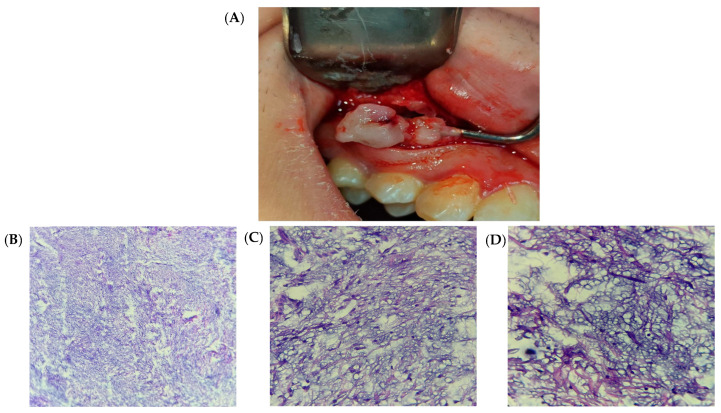
Histopathological findings of the maxillary odontogenic myxoma. (**A**) Incisional biopsy specimen of the lesion showing whitish, translucent, and mucinous appearance. (**B**) Low-power magnification (5× showing a prominent, loose, and hypocellular myxoid stroma. (**C**) Medium-power magnification (10×) revealing the characteristic spindle-shaped and stellate cell morphology along with hyperchromatic nuclei. (**D**) High-power magnification (40×) demonstrating detailed spindle-shaped neoplastic cells with delicate cytoplasmic processes completely immersed within the abundant myxoid extracellular matrix.

**Figure 3:**
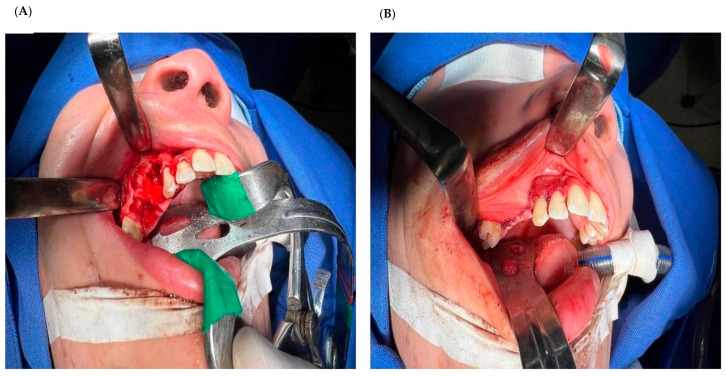
(**A**) Intraoperative image during enucleation and curettage of the odontogenic myxoma (OM). (**B**) Intraoperative image showing closure of the surgical bed.

**Figure 4:**
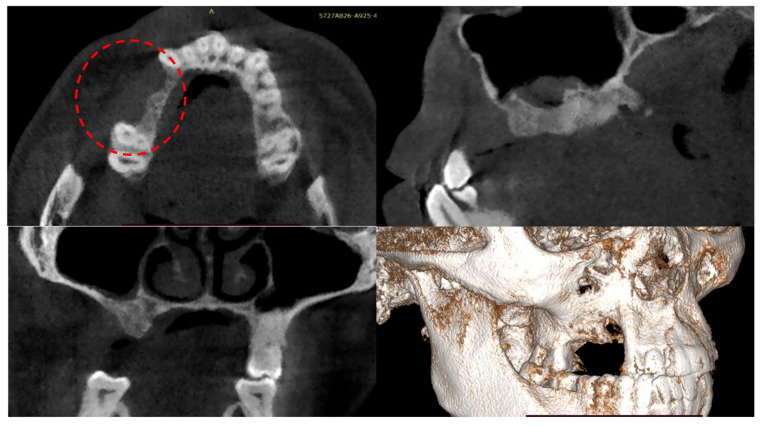
CBCT of the right maxilla at 6 months post-surgery, showing no lesion recurrence and light new bone formation. 3D reconstruction shows the defect in the right maxilla. Red dashed lines indicate light new bone formation.

**Figure 5:**
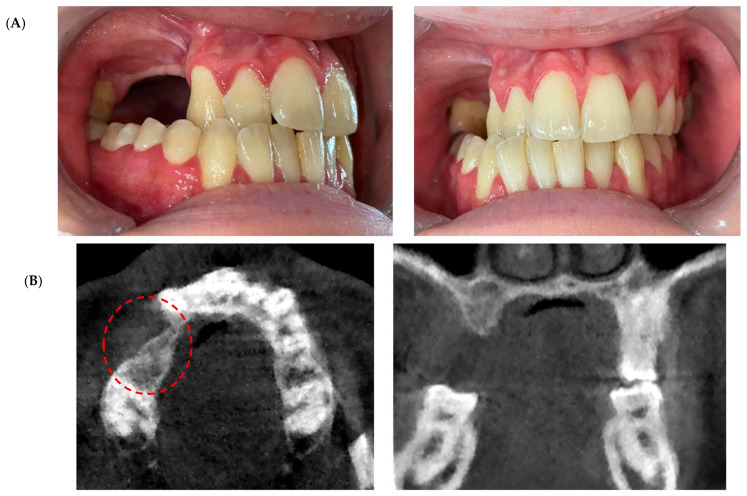
(**A**) Intraoral image of the right posterior maxilla 10 months after surgery. (**B**) CBCT of the posterior right maxilla 10 months postoperatively, showing no lesion recurrence. Red dashed lines indicate new bone formation within the osseous defect.

**Table 1:** Timeline of clinical events, diagnostics, and management.

Date/Period	Clinical Events & Diagnostic Interventions	Therapeutic Interventions & Follow-Up
**Initial Presentation**	Patient presented with a history of localized dental infections and a progressively growing maxillary mass. Concomitant history of atopic dermatitis and recent COVID-19 infection (managed with high-dose corticosteroids).	Comprehensive systemic evaluation and initial pain management.
**Diagnostic Workup**	Advanced imaging (CT scans) revealed a well-defined osteolytic lesion in the maxilla. Incisional biopsy performed.	Histopathological examination (H&E staining) confirmed the definitive diagnosis of maxillary odontogenic myxoma.
**Surgical Treatment**	Preoperative multidisciplinary clearance completed.	Surgical treatment consisting of conservative enucleation followed by meticulous peripheral curettage.
**10-Month Follow-up**	Routine clinical and radiographic evaluation.	Confirmed initial bone healing and stable functional outcomes. No signs of early recurrence.
**24-Month Follow-up**	Long-term clinical and radiographic surveillance.	Documented excellent bone regeneration and stable structural outcomes. No clinical or radiographic evidence of tumor recurrence at 24 months post-surgery.

## Data Availability

The original contributions presented in this study are included in the article. Further inquiries can be directed to the corresponding authors.

## Abbreviations

The following abbreviations are used in this manuscript:

OM	Odontogenic myxoma
WHO	World Health Organization
CT	Computed tomography
CBCT	Cone-beam computed tomography
